# Supplementary material for: Antimicrobial use through consumption of medicated feeds in chicken flocks in the Mekong Delta of Vietnam: A three-year study before a ban on antimicrobial growth promoters
Source: PLoS One. 2021 Apr 22;16(4):e0250082. doi: 10.1371/journal.pone.0250082 (PMC8061946; doi:10.1371/journal.pone.0250082)
Supplement: S1 Table — (DOCX) [file pone.0250082.s001.docx]

**S1 Table.** Detailed information on antimicrobial-containing feed formulations intended for chickens.

| No | Feed product code | Stage of production | No of AAIs | Formulation | AAIs concentration (mg/kg of feed) | | | | | | | | Type of labelling | No. of farms | No. of flocks |
| --- | --- | --- | --- | --- | --- | --- | --- | --- | --- | --- | --- | --- | --- | --- | --- |
|  |  |  |  |  | Avilamycin | Bacitracin | Chlortetracycline | Colistin | Enramycin | Flavomycin | Oxytetracyline | Virginimycin |  |  |  |
| 1 | FE001 | Brooding | 1 | Crumb |  |  |  |  | 10 |  |  |  | Certain | 42 | 127 |
| 2 | FE002 | Growing | 1 | Pellet |  |  |  |  | 10 |  |  |  | Certain | 9 | 11 |
| 3 | FE004 | Growing | 1 | Pellet |  |  |  |  | 10 |  |  |  | Certain | 37 | 109 |
| 4 | FE005 | Brooding | 1 | Crumb |  |  | 50 |  |  |  |  |  | Certain | 6 | 13 |
| 5 | FE006 | Growing | 1 | Pellet |  |  | 50 |  |  |  |  |  | Certain | 4 | 10 |
| 6 | FE007 | Brooding | 1 | Pellet |  |  | 50 |  |  |  |  |  | Certain | 8 | 19 |
| 7 | FE011 | Brooding | 1 | Crumb |  |  | 50 |  |  |  |  |  | Certain | 4 | 6 |
| 8 | FE013 | Growing | 1 | Pellet |  |  | 10-50 |  |  |  |  |  | Certain | 1 | 2 |
| 9 | FE015* | Brooding | 1 | Crumb |  |  | 50 | 50* |  |  | 50 |  | Ambiguous | 3 | 4 |
| 10 | FE017 | Finishing | 1 | Pellet |  | 50 |  |  |  |  |  |  | Certain | 1 | 1 |
| 11 | FE019 | Brooding | 1 | Crumb |  | 30 |  |  |  |  |  |  | Certain | 27 | 79 |
| 12 | FE023 | Brooding | 1 | Crumb |  |  |  |  | 10 |  |  |  | Certain | 2 | 2 |
| 13 | FE024 | Brooding | 1 | Crumb |  |  | 50 |  |  |  |  |  | Certain | 13 | 26 |
| 14 | FE025 | Growing | 1 | Pellet |  |  | 50 |  |  |  |  |  | Certain | 9 | 24 |
| 15 | FE026 | Finishing | 1 | Pellet | 10 | 50 |  |  | 10 | 10 |  |  | Ambiguous | 3 | 5 |
| 16 | FE033 | Brooding | 1 | Crumb | 10 | 50 |  |  | 10 | 10 |  |  | Ambiguous | 2 | 2 |
| 17 | FE036 | Growing | 1 | Pellet |  |  |  |  | 1-10 |  |  |  | Certain | 5 | 5 |
| 18 | FE037 | Brooding | 1 | Pellet |  |  | 50 |  |  |  |  |  | Certain | 7 | 16 |
| 19 | FE040 | Brooding | 1 | Mask |  | 50 |  |  |  |  |  |  | Certain | 1 | 3 |
| 20 | FE042 | Brooding | 1 | Pellet |  |  | 50 |  |  |  |  |  | Certain | 1 | 1 |
| 21 | FE043 | Growing | 1 | Pellet |  |  | 50 |  |  |  |  |  | Certain | 1 | 1 |
| 22 | FE045 | Finishing | 1 | Pellet | 10 | 50 |  |  | 10 | 10 |  |  | Ambiguous | 1 | 1 |
| 23 | FE047 | Brooding | 1 | Crumb |  | 50 |  |  | 10 |  |  |  | Ambiguous | 3 | 3 |
| 24 | FE048 | Growing | 1 | Pellet |  |  |  |  | 10 |  |  |  | Certain | 3 | 3 |
| 25 | FE053 | Brooding | 1 | Pellet |  | 50 |  |  | 10 |  |  |  | Ambiguous | 2 | 5 |
| 26 | FE055* | Finishing | 1 | Pellet |  | 50 |  |  | 15* |  |  | 5 | Ambiguous | 1 | 4 |
| 27 | FE058 | Brooding | 1 | Pellet |  | 4-50 |  |  | 1-10 |  |  |  | Ambiguous | 2 | 2 |
| 28 | FE059 | Brooding | 1 | Crumb |  |  |  |  |  |  |  | 5-15 | Certain | 6 | 6 |
| 29 | FE066* | Growing | 1 | Pellet | 15 | 125* |  |  | 10 | 2 |  |  | Ambiguous | 2 | 2 |
| 30 | FE067 | Brooding | 1 | Crumb |  |  | 50 |  |  |  |  |  | Certain | 1 | 2 |
| 31 | FE074* | Brooding | 1 | Crumb | 15 | 125* |  |  | 10 | 2 |  |  | Ambiguous | 4 | 4 |
| 32 | FE080 | Brooding | 1 | Crumb |  |  |  |  | 1-10 |  |  |  | Certain | 1 | 2 |
| 33 | FE090* | Finishing | 1 | Pellet |  | 50-100* |  | 60-160* |  |  |  |  | Ambiguous | 2 | 2 |
| 34 | FE094* | Brooding | 1 | Crumb |  | 50-100* |  | 60-160* |  |  |  |  | Ambiguous | 1 | 1 |
| 35 | FE104* | Brooding | 2 | Crumb |  |  | 75-150* | 100-200* |  |  |  |  | Certain | 1 | 1 |

*Products with AAIs concentration not permitted under Vietnamese regulations.
